# Synthesis, insertion, and characterization of SARS-CoV-2 membrane protein within lipid bilayers

**DOI:** 10.1126/sciadv.adm7030

**Published:** 2024-02-28

**Authors:** Yuanzhong Zhang, Sara Anbir, Joseph McTiernan, Siyu Li, Michael Worcester, Pratyasha Mishra, Michael E. Colvin, Ajay Gopinathan, Umar Mohideen, Roya Zandi, Thomas E. Kuhlman

**Affiliations:** ^1^ Department of Physics and Astronomy, University of California, Riverside, Riverside, CA 92521, USA.; ^2^ Biophysics Program, University of California, Riverside, Riverside, CA 92521, USA.; ^3^ Department of Physics, University of California, Merced, Merced, CA 95340, USA.; ^4^ Department of Biochemistry, University of California, Riverside, Riverside, CA 92521, USA.; ^5^ Department of Chemistry and Biochemistry, University of California, Merced, Merced, CA 95340, USA.; ^6^ Microbiology Program, University of California, Riverside, Riverside, CA 92521, USA.

## Abstract

Throughout history, coronaviruses have posed challenges to both public health and the global economy; nevertheless, methods to combat them remain rudimentary, primarily due to the absence of experiments to understand the function of various viral components. Among these, membrane (M) proteins are one of the most elusive because of their small size and challenges with expression. Here, we report the development of an expression system to produce tens to hundreds of milligrams of M protein per liter of *Escherichia coli* culture. These large yields render many previously inaccessible structural and biophysical experiments feasible. Using cryo–electron microscopy and atomic force microscopy, we image and characterize individual membrane-incorporated M protein dimers and discover membrane thinning in the vicinity, which we validated with molecular dynamics simulations. Our results suggest that the resulting line tension, along with predicted induction of local membrane curvature, could ultimately drive viral assembly and budding.

## Teaser

High-yield purification of SARS-CoV-2 M protein enables characterization of interactions driving virus formation.

## INTRODUCTION

Coronaviruses have caused severe health crises and economic challenges over the centuries. Although the likelihood of another coronavirus type emerging after the eradication of one has always been high, research on them has often diminished due to limited success in understanding fundamental elements of their functioning and life cycle. This is in sharp contrast to progress on other viruses like HIV and hepatitis B virus. One of the most notable factors contributing to our limited knowledge of coronaviruses is the difficulty in performing in vitro experiments on viral proteins due to challenges posed by the small size of the proteins and low yield expression methods for them. As an example, while the membrane (M) protein of severe acute respiratory syndrome coronavirus 2 (SARS-CoV-2) is the most abundant protein with a crucial role in the formation of the virus, it is much less well characterized than most other SARS-CoV-2 structural proteins. Here, we take important steps toward developing a deeper understanding of M protein structure and function in the physiologically relevant membrane environment by developing an expression technique with orders of magnitude higher yield, inserting the protein into lipid membranes and characterizing the protein-membrane configurations using a combination of atomic force microscopy (AFM), cryo–electron microscopy (cryo-EM), and molecular dynamics (MD) simulations.

Specifically, using a small ubiquitin-related modifier (SUMO) tag–based expression system, we produced and purified substantial quantities of full-length, native M protein in *Escherichia coli*. As far as we are aware, this is the only reported method for producing substantial quantities of full-length, native M protein, and our method is of low cost and produces large quantities of pure native, full-length protein with low endotoxin levels. We examined the purity of our M protein product using Western blot and matrix-assisted laser desorption/ionization (MALDI) mass spectrometry and showed that it has low endotoxin activity. Our method produces tens to hundreds of milligrams of protein per liter of culture, with these high yields enabling many previously inaccessible experiments, including small-angle x-ray scattering and cryo-EM.

In addition, we developed a reliable method for inserting purified M protein dimers into a suspended lipid membrane in a homogeneous orientation, and we used cryo-EM and AFM to image the M protein dimers in the membrane. We compared our experimental results to all-atom MD simulations of the M protein dimers in a lipid membrane to provide structural information about the protein and the surrounding membrane to aid in interpreting the imaging results. While some prior experimental studies (*1*) revealed two distinct M protein structures, a “short” and a “long” form, other studies only showed the existence of a single–M protein structure (*2*), similar to the short form. Our AFM results, together with our atomistic MD simulations, indicate that M protein dimers within our supported lipid bilayer are entirely in the short form identified in earlier structural studies.

Our AFM results also uncovered a thinning of the membrane in the vicinity of the M protein dimer, which we confirmed with MD simulations. In addition, our MD simulations indicated the propensity of individual M protein dimers to induce local membrane curvature. The line tension resulting from the thinning together with the induced curvature could potentially drive the aggregation viral proteins and the subsequent budding of the virion. To test this idea, we constructed a coarse-grained simulation model of M proteins in a flat membrane and showed that endodomain interactions among M protein dimers were also critical for budding. Together, our work establishes a framework for future studies of viral protein and sheds light on the structure and interactions of M protein dimers within a lipid membrane, yielding insights into important physical processes that drive viral assembly and budding.

## RESULTS

### Expression and high-yield purification of SARS-CoV-2 M protein

**Fig. 1.**
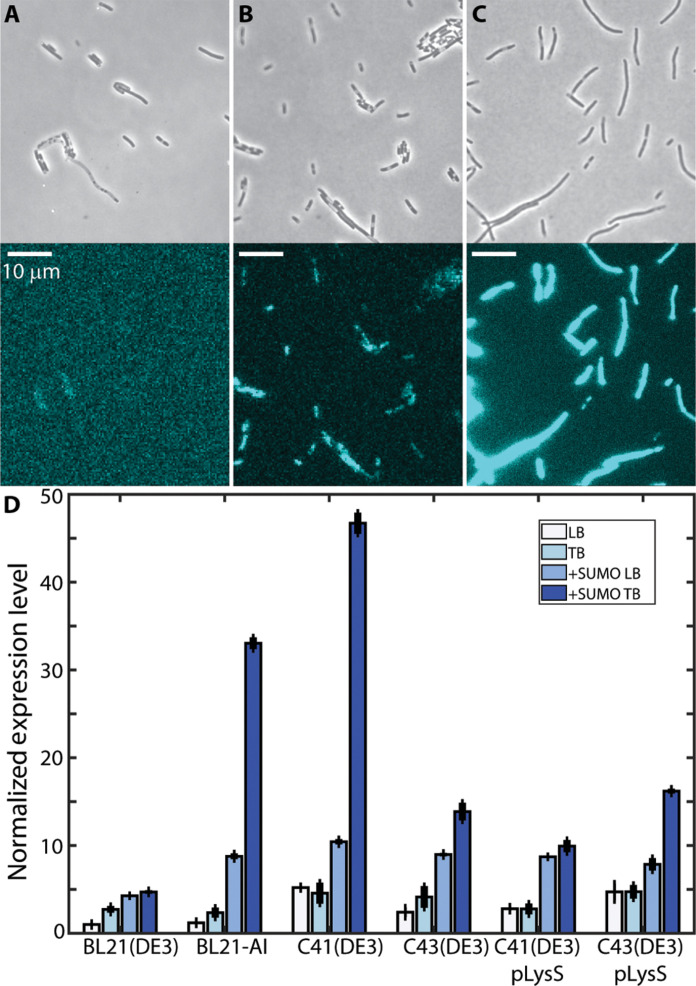
Membrane protein expression in *E. coli*. (**A** to **C**) Fluorescence microscopy of M-mCerulean3 expression in terrific broth (TB) medium, with phase contrast image shown above and mCerulean3 fluorescence channel shown below. (A) M-mCerulean3 expressed in BL21(DE3). (B) SUMO-M-mCerulean3 expressed in BL21(DE3). (C) SUMO-M-mCerulean3 expressed in C41(DE3). All strains were imaged with identical microscope and camera settings, and images are displayed with identical lookup tables. Scale bars, 10.0 μm. (**D**) Quantitative comparison of M-mCerulean3 expression in various *E. coli* strains and media [white, lysogeny broth (LB); light blue, TB; SUMO-M-mCerulean3 in LB, medium blue; SUMO-M-mCerulean3 in TB, dark blue]. Expression levels are normalized to expression of M-mCerulean3 in BL21(DE3).

To assess the level of expression of SARS-CoV-2 M protein in *E. coli*, we constructed a C-terminal fusion of M with the blue fluorescent protein mCerulean3. This fusion protein is borne on the high–copy number plasmid pUC57 and expressed from the inducible promoter P*lac*T7. Upon transformation into the standard expression strain BL21(DE3), we observed extremely low expression regardless of the degree of induction with isopropyl β-d-thiogalactopyranoside (IPTG) (Fig. 1A). Previous reports have demonstrated that expression of M protein from the original SARS-CoV virus could be substantially enhanced by N-terminal fusion of M with a SUMO tag (*3*). Following this same strategy resulted in enhancement of expression by ~4 to 5 times (Fig. 1, B and D). We further optimized yields by quantifying expression of M-mCerulean3 and SUMO-M-mCerulean3 in various strains of *E. coli*, particularly the Walker strains, C41(DE3) and C43(DE3), which have been specifically selected for high expression of membrane-bound and other difficult proteins. We measured expression in these strains grown in both lysogeny broth (LB) and terrific broth (TB), finding that expression of SUMO-M-mCerulean3 in C41(DE3) grown in TB is ~45 to 50 times higher than that of the standard use of BL21(DE3) grown in LB.

**Fig. 2.**
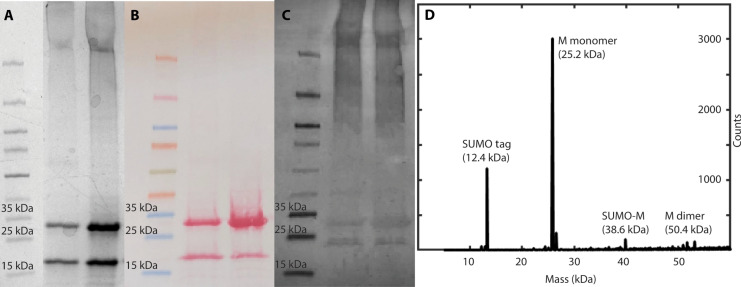
Verification of purified M protein. (**A**) SDS–polyacrylamide gel electrophoresis (PAGE) of purified M sample. Lane 1, ladder; lane 2, after digestion with 0.5 mg SUMO protease per milligram of M; lane 3, digestion with 2× amount SUMO protease. (**B**) Ponceau all-protein stain of protein transferred to blot from PAGE gel. Lanes same as (A). (**C**) Western blot of (A). (**D**) MALDI mass spectrum of purified M sample.

Purification of SUMO-M from C41(DE3) grown in TB using standard immobilized metal affinity chromatography (IMAC) resulted in high yields, typically on the order of 50 to 100 mg per liter of culture. Following purification, we refolded the protein by dialysis and removed the 6xHis tag and SUMO tag by digestion with Ulp1 SUMO protease, resulting in pure, full-length, native M protein. We quantified the purity by SDS–polyacrylamide gel electrophoresis (PAGE), Western blot, and MALDI (Fig. 2) and quantified endotoxin levels by Limulus amebocyte lysate (LAL) endotoxin assay [0.35 ± 0.05 endotoxin units (EU)/mg]. SDS-PAGE (Fig. 2A) and MALDI mass spectrometry (Fig. 2D) show residual cleaved 6xHis-SUMO tag (12.4 kDa), which was subsequently removed by further IMAC purification. Furthermore, Western blot shows (Fig. 2, B and C), in addition to a large band at the predicted M monomer molecular weight of 25.2 kDa, a large streak of high–molecular weight multimers and aggregates, as well as a small amount of lower–molecular weight products that may be degradation products. However, the majority of detectable products are M monomer and aggregates thereof.

We emphasize that, while *E. coli* is unable to perform posttranslational modifications, such as glycosylation that is predicted to occur posttranscriptionally (*4*), it is known for the related SARS-CoV virus that these modifications to M protein are not required for viral assembly and do not substantially impact protein-protein interactions (*5*–*8*).

### Cryo-EM of lipid bilayer membrane reconstituted with M protein in LUVs

**Fig. 3.**
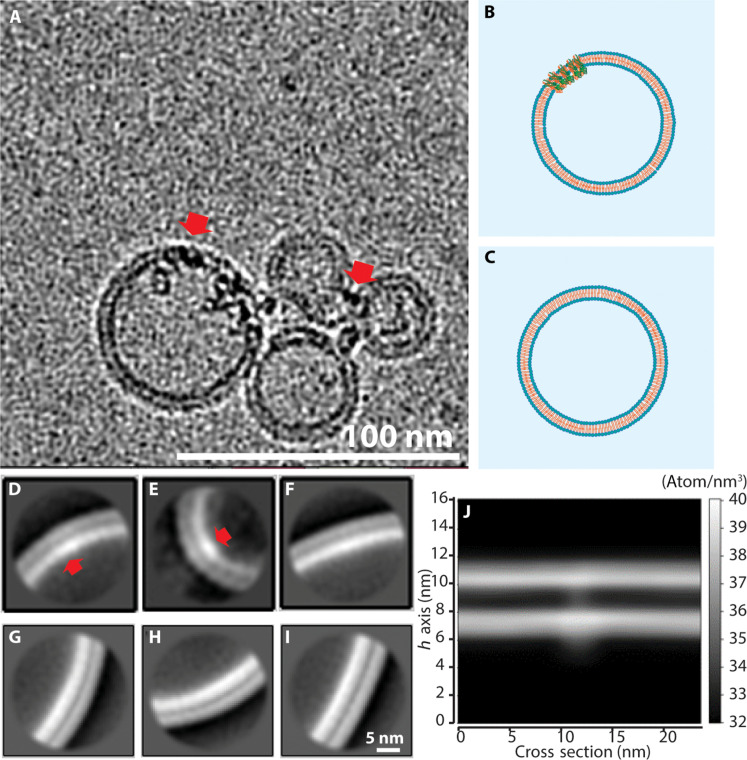
Typical cryo-EM micrographs of LUVs reconstituted with M proteins. (**A**) Red arrows indicate M proteins inserted in the vesicle membrane. (**B** and **C**) Schematics of LUVs with and without M reconstitution, respectively. (**D**) Representative two-dimensional (2D) class average image of areas without M protein in reconstituted LUVs. (**E** and **F**) Two most representative cryo-EM 2D class average images from LUV bilayer membranes reconstituted with M protein mass ratio, *R*_m,M/L_ = 0.015. Arrows, location of M protein as bright spots. (**G** to **I**) 2D class average cryo-EM images of blank LUV bilayer membrane. Scale bar, 5.0 nm. (**J**) Atom number density of the short conformation M protein embedded in a bilayer membrane obtained from all-atom MD.

Next, to facilitate studies of the M protein in its physiologically relevant context, we developed a procedure for M protein insertion into lipid membranes of large unilamellar vesicles (LUVs) with a phospholipid composition mimicking that of a typical endoplasmic reticulum–Golgi intermediate compartment (ERGIC) bilayer membrane [described in detail in Materials and Methods and in the Supplementary Materials (fig. S1)]. We then used cryo-EM and AFM to verify the presence of the inserted M protein and its orientation, as described below. First, cryo-EM studies of LUVs with and without M protein were done to confirm the presence of M proteins in the bilayer membranes. A representative cryo-EM image of the M protein embedded in bilayer vesicles is shown in Fig. 3A, with the schematic of LUVs with (Fig. 3B) and without (Fig. 3C) M protein embedded. Figure 3 (D to F) shows the typical two-dimensional (2D) class average images of cryo-EM observations from LUVs reconstituted with M protein at a protein-to-lipid mass ratio of 0.015. Two types of 2D class averages were observed in the cryo-EM images. Figure 3 (D and E) shows the M protein as bright spots on the bilayer corresponding to higher electron density of the M protein embedded in the membrane, while, in the image in Fig. 3F, there is no M protein embedded. Most frequently observed spots were ~4- to 5-nm wide, which can be associated with single dimers or possibly some higher order oligomers. We also observed that these spots corresponding to the M protein show a higher intensity on the inner leaflet of the vesicle, indicating that they are likely inserted with the larger and denser C-terminal facing the inside of vesicles. In contrast with regions reconstituted with M proteins, the regions without M proteins show homogeneous intensity from the corresponding uniform electron density throughout the bilayer membrane. In the bottom row as a negative control, blank vesicle samples without M protein were also imaged with cryo-EM. Figure 3 (G to I) shows typical 2D class average images with a homogenous electron density observed for the LUVs without M protein. Last, Fig. 3J shows a simulation of these cryo-EM micrographs with M protein calculated from the MD simulations of the short form of M protein embedded in a membrane by projecting the atom number density onto a plane perpendicular to the surface of the membrane using a bin size of 0.95 nm by 0.95 nm. Schematics for this MD simulation are shown in fig. S2, with details provided in Materials and Methods. Two different viewing angles are averaged over in creating this simulated image, where lower-resolution density maps for both the long and short forms are shown in fig. S3.

### AFM topography of SBL reconstituted with embedded M protein

**Fig. 4.**
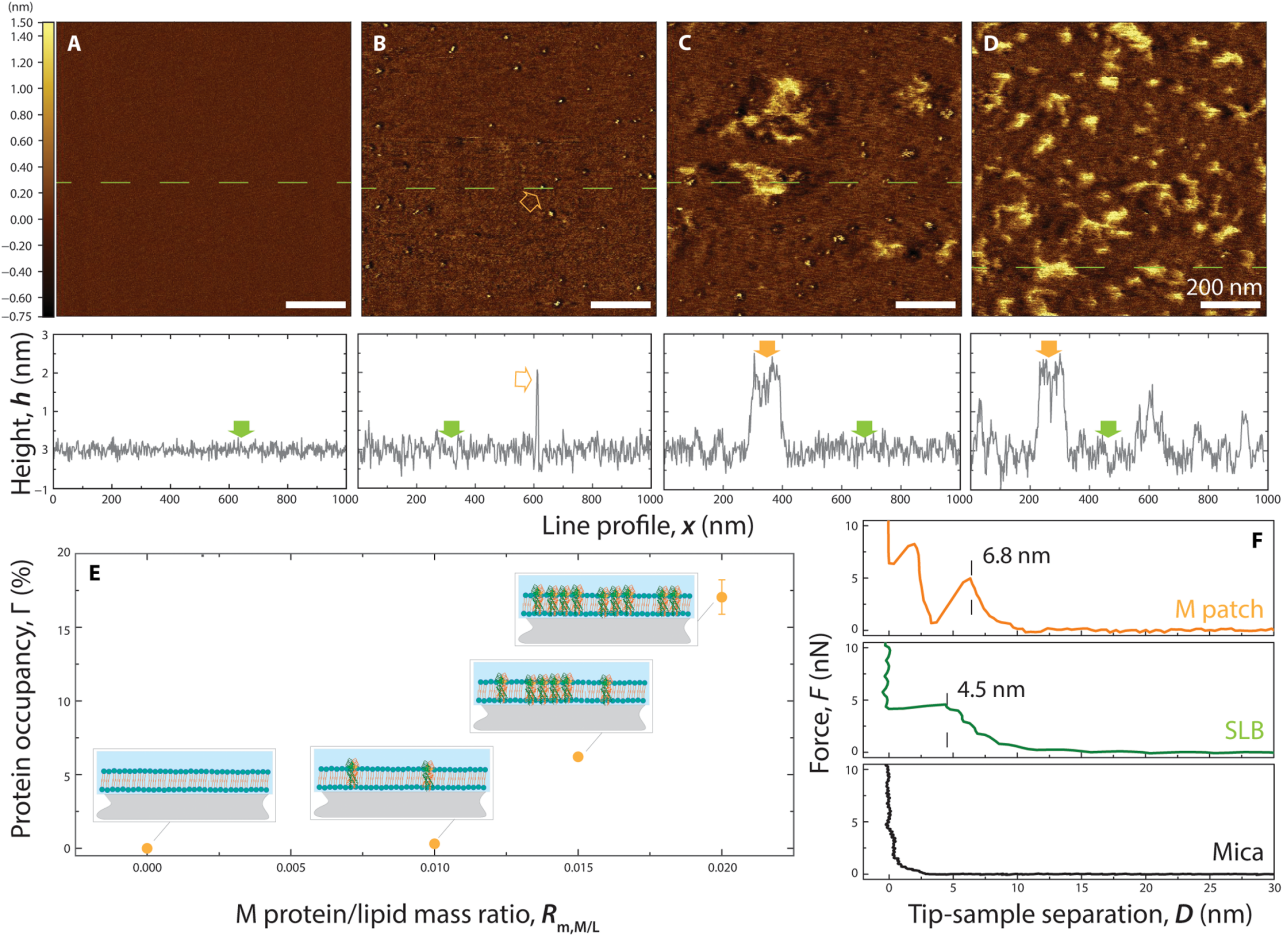
AFM height image of SBL reconstituted with M protein at increasing protein-to-lipid mass ratios, *R*_m,M/L_, imaged on mica substrates. (**A** to **D**) Height images and representative single line profiles (green dashed lines in images) collected from supported bilayer (SBL) fabricated from LUVs that were reconstituted with M protein at *R*_m,M/L_ = 0, 0.01, 0.015, and 0.02. (**E**) Area occupation percentage of M protein in SBL as a function of *R*_m,M/L_ calculated from (A) to (D). (**F**) Representative force profiles collected from pristine mica surface (black), SBL (green), and M protein (orange) patches in lipid bilayer; the force profiles were collected during tip engagement.

While the cryo-EM results presented above indicated the presence of the M protein in the lipid bilayer, the small molecular weight of 50.2 kDa for the dimer limits high-resolution analysis of the M protein–lipid membrane interaction. To confirm the cryo-EM results, we used the AFM with the height resolution of 0.01 nm and a lateral resolution 0.8 nm, which is ideally suited for understanding the inserted protein morphology, orientation, oligomerization at larger concentration, and the protein-lipid bilayer interaction (*9*, *10*). Figure 4A shows a 1 μm–by–1 μm AFM topography of a typical ERGIC bilayer membrane generated from LUVs without M protein reconstitution. The height profile along the green dashed line is shown in the bottom panel of Fig. 4A. The supported bilayer (SBL) on the mica substrate is smooth with root mean square roughness measured to be 0.3 nm. Figure 4B shows the typical topography of a 1 μm–by–1 μm surface of a SBL generated from LUVs reconstituted with M protein at a protein-to-lipid mass ratio of 0.01. In contrast to the smooth surface of the SBL in Fig. 4A, scattered single-particle protrusions were observed in the AFM image. These can be associated with the embedded protein in the SBL. The line profile (green dashed line) passing through one of the M proteins shows that the height of the feature above the membrane is 2.4 nm, consistent with the C-terminal height of M protein from literature (*1*, *2*) and simulations presented below. Figure 4 (C and D) shows the SBL from LUVs reconstituted at higher protein-to-lipid mass ratios of 0.015 and 0.02, respectively. The topography in Figure 4C shows a combination of scattered single particles and patches, while patches are mostly observed in Fig. 4D. The line profiles show that the patches have the same height as the single particles associated with the M protein. Figure 4E shows the percent of protein-occupied area in the SBL membrane as a function of protein-to-lipid ratio. The area occupied by the protein is calculated from the sum of the single protein and patch areas. The M protein–occupied area increases from 0.3 to 6.2 and 17.1%, as the protein ratio increased from 0.01 to 0.015 and 0.02, respectively.

In addition to examining the planar surface features of the SBL with and without M protein, we also characterized the depth profile using AFM indentation studies. Figure 4F shows a typical indentation force profile obtained from three different samples: bare mica in the bottom panel; SBL without and with M protein (concentration ratio of *R*_m,M/L_ = 0.02) in the middle and top panels, respectively. In all three panels of Fig. 4F, the horizontal axis is the separation distance between the AFM probe tip and the bare mica surface. The bottom panel shows the typical indentation force curve of the bare mica surface in solution with the tip-rigid surface contact at zero followed by a sharp increase in force by the cantilever bending. The middle panel corresponds to the SBL membrane without M protein. Here, the AFM tip comes in contact with the hydration layer at a separation around 7.5 nm with the applied force leading to deformation of the membrane. With increasing applied force, the tip punctures the membrane surface at *D* = 4.5 ± 0.1 nm. This corresponds to the membrane thickness without M protein. With further increase in force, the AFM tip penetrates the membrane until it contacts the rigid mica at 0 nm. The top panel shows the indentation force profile obtained from the patch-like structures from the M protein on the SBL. The force showed a discontinuity at 6.1 nm which corresponds to the tip-protein surface interaction. Large forces lead to penetration of the tip into the M protein patch. The discontinuity at 3.2 nm indicates potential disruption of a specific strong interaction region between neighboring M proteins. From the slope of the approach curves in Fig. 4F, the Young’s modulus can be calculated to be 9.5 MPa on the lipid bilayer and 41.5 MPa on the protein patches, respectively. Figure S4 shows the average elastic moduli extracted from nanoindentation force profiles for SBL and the M protein patches. The difference in the Young’s modulus indicates that the patches are a different type of material from the lipid bilayer.

### Formation of M protein aggregate patches in the SBL

From Fig. 4B, we observed that M proteins in SBL start as single–M protein assemblies at an M protein–to–lipid mass concentration *R*_m,M/L_ = 0.01. From the size (length, width, and height), they can be identified as M protein dimers. With the increase of M protein concentration in the SBL, the dimers aggregate to form patches of M proteins as observed in Fig. 4 (C and D). As seen in Fig. 4E, the area of the patches grows nonlinearly with the M protein concentration. The M protein–occupied area increases from 0.3 to 6.2 and 17.1%, as the protein-to-lipid mass ratio *R*_m,M/L_ increased from 0.01 to 0.015 and 0.02. Thus, the M protein incorporation into the membrane is facilitated by the presence of M proteins already in the membrane. Notice that the height of patch-like assemblies is the same as the isolated single–M protein dimers observed at the lower M concentration. This indicates that the M proteins assemble in a side-by-side manner inside the bilayer, as demonstrated by schematics shown in Fig. 4E; these patches are higher order oligomers.

### Morphological statistics of the isolated M protein particle

**Fig. 5.**
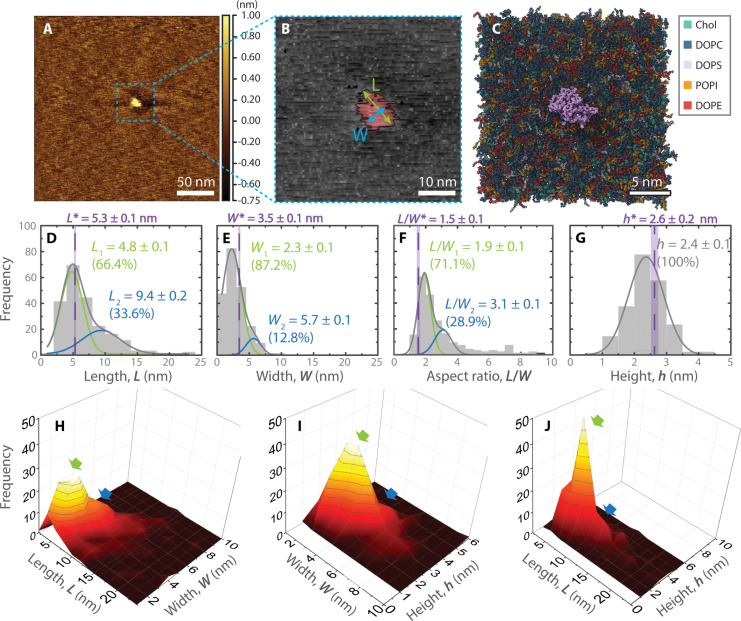
Size and morphology statistics of M protein quantified from AFM images of SBL. (**A**) Representative AFM height image of individual M protein reconstituted in SBL at *R*_m,M/L_ = 0.01. (**B**) Zoomed-in image of (A), highlighting the protein with red color and lipid bilayer with gray color. The protruded portion of a single–M protein particle above lipid bilayer (left) is detected by setting the height of top surface of SBL as a threshold and taking the above portion. (**C**) Top view of the short conformation M protein (C terminus in purple) embedded in a multicomponent membrane after 1 μs into an all-atom MD simulation. Dimensional parameters—length (*L*), width (*W*), and height (*h*)—were measured for detected protein particles. (**D** to **G**) Histograms of *L*, *W*, *L*/*W*, and *h* of protein protruding above lipid membrane. Solid lines are Gaussian fits. Vertical dashed lines mark corresponding values from MD simulation for short-form M protein reconstituted with C terminus exposed above SBL. (**H** to **J**) 3D histograms each showing combined statistical survey of two out of the three dimensions. Green and blue arrows indicate dimer and higher order of oligomers, respectively.

We next characterized the properties of single–M protein particles embedded in the SBL for protein-to-lipid mass ratio, *R*_m,M/L_ = 0.01 (Fig. 5). Figure 5A shows a high-resolution 250 nm–by–250 nm surface morphology of a typical single isolated M protein at the concentration ratio of *R*_m,M/L_ = 0.01. In Fig. 5B, the characterization parameters the length (*L*) and width (*W*) are shown. The boundary of the protein particle is identified as an edge higher than the planar membrane height. The planar membrane height is found by averaging the membrane height far away from the particle (gray region, Fig. 5B). The particle thus identified is shown in red in Fig. 5B. The length (*L*) of the particle is defined as the maximum distance between any two points on the perimeter of the protein particle. The particle width (*W*) is defined as the maximum distance between two points on the perimeter, in the direction perpendicular to length. The particle height (*h*) is the height of the highest point on the protein surface. For comparison, Fig. 5C shows the final frame of an all-atom MD simulation for the short conformation M protein embedded in a multicomponent membrane (see fig. S2 for schematics). The analysis of the AFM data is repeated for 214 samples, and the histogram of the results for *L*, *W*, *h*, and the ratio *L*/*W* are shown in Fig. 5 (D to G). Corresponding size classifications from MD simulations are shown in Fig. 5 (D to G), averaged over the last 100 ns of the simulation, through the purple dashed lines (see figs. S5 to S7 for more information). Protein length and width were obtained by fitting an ellipse to the projection of the protein above the membrane, while height was based on the difference between the top of the M protein C-terminal and the phospholipid heads adjacent to the protein. The histogram in Fig. 5D shows a bimodal distribution, which, when fit to two normal distributions, leads to mean particle lengths, *L*_1_ = 4.8 ± 0.1 nm and *L*_2_ = 9.4 ± 0.2 nm. Figure 5E shows the width distribution, again seen to be bimodal, which, when fit to two normal distributions, leads to mean values of *W*_1_ = 2.3 ± 0.1 nm and another at *W*_2_ = 5.7 ± 0.1 nm. In Fig. 5F, the aspect ratio *L*/*W* again shows two normal distributions: one at *L*/*W*_1_ = 1.9 ± 0.1 and the other at *L*/*W*_2_ = 3.1 ± 0.1. Figure 5G shows the histogram obtained from the height of the M protein particles. The values can be fit to one normal distribution with a mean height *h* = 2.4 ± 0.1 nm.

### Confirmation of M protein insertion in the bilayer membrane

High-resolution cryo-EM studies have shown that SARS-CoV-2 membrane protein dimers exist in a conformational equilibrium between the long form and the short form, which are 7.2 and 8.6 nm in height, respectively (*1*). Comparing these heights with those in Fig. 5G, AFM shows that the height of exposed protein particles is much shorter (2.4 nm). This is in agreement with the all-atom MD simulations, where the height ranges from 2.6 to 2.8 nm depending on the protein conformation (short/long, see fig. S5B). The height measured by AFM is more consistent with the short-form height from simulations; a more thorough discussion of these height fluctuations can be found in the Supplementary Materials (fig. S8). The slight differences between AFM and MD can be attributed to the protein deforming slightly as the AFM tip makes contact. Meanwhile, the lateral dimensions of isolated protein particles observed from AFM in Fig. 5 (D and E) are very similar to the literature reported values of 5.0 and 5.7 nm for long and short forms, respectively (*1*). A much smaller height and similar lateral dimensions indicate that the M proteins are inserted into the bilayer membranes instead of absorbing on top of the membranes. This is also supported by our cryo-EM observations in Fig. 3 (D and E). The membrane reconstituted with M protein shows a higher electron density compared with blank membrane. This indicates that a large portion of the protein is buried inside the bilayer membrane.

### Orientation of reconstituted M protein with C terminus facing the vesicle interior

In Fig. 3 (D and E), we observed that the bright spots corresponding to reconstituted proteins show a higher electron density in the inner leaflets. This indicates that the C terminus, which is larger than the N terminus and contains a higher density of electron-rich “heavy” atoms, is inserted facing the inside of the vesicles. This is further confirmed in AFM measurements and also matches the simulated cryo-EM image from MD in Fig. 3J. This stems from averaging the protein over different angles, because the N terminus of the protein on the outer leaflet ranges from small to large arc length along the membrane. Alternatively, the C-terminal domain of the protein along the inner leaflet is largely independent of the viewing angle and will have greater arc length than the N-terminal domain (see fig. S3 for viewing angle differences). In Fig. 5G, the height of reconstituted M protein exposed above bilayer membrane show a single distribution with an average height of 2.4 nm, which is consistent with C-terminal size observed in high-resolution cryo-EM images and our simulation predictions, indicating that all M proteins in the SBLs observed in AFM are oriented with their C termini facing the AFM probe. From these results, we can deduce that the M proteins were reconstituted in LUVs with their C terminus encapsulated inside the vesicles. This is because, during the formation of the SBL on mica, the LUVs adsorb to the mica surface with their outer leaflets. Subsequently, the LUVs rupture, exposing their inner leaflets facing upward and accessible to the AFM probe.

### Reconstituted M protein dimensions and membrane thickness consistent with the short form

In Fig. 5 (H to J), the three-dimensional histograms analyze the relationships between protein length (*L*) and width (*W*), width and height (*h*), as well as length and height, respectively. In Fig. 5H from the *L* and *W*, we can observe two distinct populations with their individual lengths and widths. From Fig. 5I plot of the *W* and *h*, we can observe that both *W* populations have the same height above the membrane. From Fig. 5J, the same is observed for the two height populations. Combining the above information with results shown in the previous two-dimensional histograms (Fig. 5, D to G), the most frequently observed M protein population in the SBL have a length, width, and height of 4.8 ± 0.1 nm, 2.3 ± 0.1 nm, and 2.4 ± 0.1 nm, respectively. These dimensions show a good agreement with the short form of M protein dimer both from published cryo-EM studies of M protein in detergent micelles (*1*) and our simulation predictions [averages shown with the purple lines in Fig. 5 (D to G); see figs. S5 to S7 for the time evolution of these quantities]. The slight discrepancy between the short conformation MD dimensions and AFM results likely stem from the original MD protein structures not including every residue and deformations in the protein from the AFM tip. The second population observed has a much larger size with a length, width, and height of 9.4 ± 0.2 nm, 5.7 ± 0.1 nm, and 2.4 ± 0.1 nm, respectively. These dimensions correspond to that of a multimer of M protein dimers.

### Reduction of membrane thickness around M protein

**Fig. 6.**
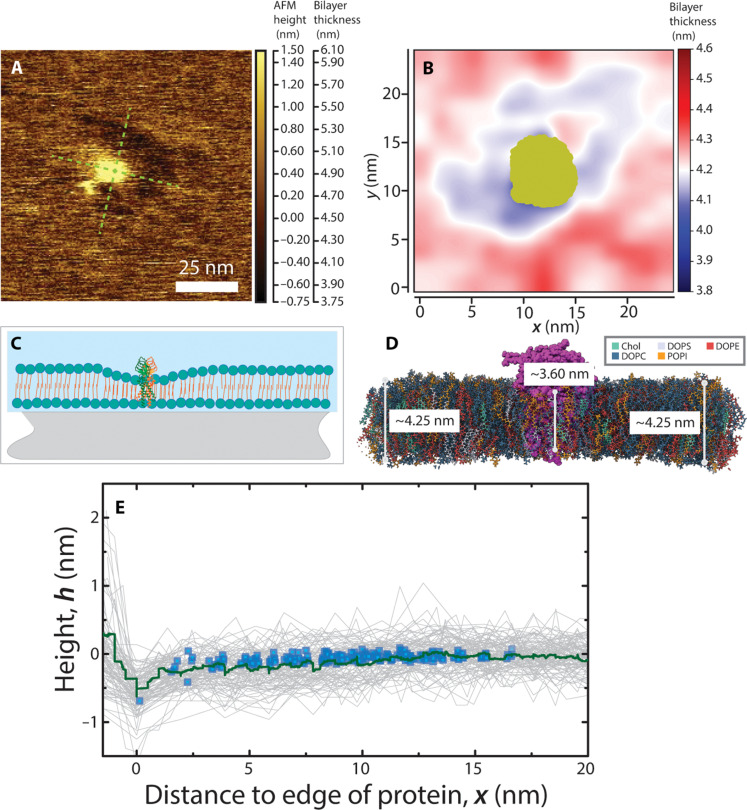
Analysis of membrane thickness around individual M protein particles at *R*_m,M/L_ = 0.01 and through MD simulation. (**A**) Example of individual M protein particles reconstituted in SBL. Height profiles were taken along the green dashed lines starting from the center of the protein. (**B**) View from above of membrane thickness in nm from 500 ns to 1 μs of an all-atom MD simulation for the short conformation M protein embedded in a multicomponent membrane. (**C**) Schematics of membrane thinning around M protein particles. (**D**) Side view of the MD simulation along the *y* axis, with the protein in purple, where average thickness is shown dependent on distance from the protein. (**E**) Solid green line shows the experimentally measured averaged height profile of membrane around individual M protein particles. Inset shows around 100 height profiles obtained from 25 individual protein particles. Here, *x* = 0 nm is defined by the boundary between the M protein particle and lipid membrane. The blue points represent vertically shifted values of membrane thickness obtained through MD shown in (B).

Figure 6A shows a typical isolated M protein particle in a magnified view, with color scale corresponding to the membrane thickness shown to the right. We observed that the membrane thickness in proximity to the single–M protein particle is reduced. The height of the membrane around each protein particle is measured along two perpendicular axes, shown in Fig. 6A as green dashed lines. A similar view from above of the M protein short conformation obtained through all-atom MD simulations can be seen in Fig. 6B, where red regions represent a thicker membrane and blue thinner (see fig. S9 for unshifted radial plot). The circular cross section of the protein shown in gold arises because of the protein rotation in the membrane during the simulation shown in fig. S10. Figure 6C shows an exaggerated diagram of the membrane thinning near the protein and a corresponding side view of the thickness after 1 μs of MD simulation is displayed in Fig. 6D. The change in membrane height from the edge of the protein (0 nm) along the dashed lines for 25 such proteins is shown in Fig. 6E. In addition, the blue points represent shifted membrane thickness values from Fig. 6B, based on the distance from the edge of the protein. These distances extend beyond half the box size as we include data from points along the diagonal, giving us a range from 0 to 16.63 nm. The mean change in height with distance from the protein edge is shown by the solid green line. We observed that the bilayer membrane is compressed until the height returns to the normal thickness of 12 nm from the edge of the protein. The maximum membrane compression at the edge of the protein has a mean value of 0.5 nm. It is to be emphasized that this AFM-determined thinning profile matches the MD short-form thinning profile, as opposed to the long form (long form shown in fig. S9) and agrees with observations of Neuman *et al.* (*11*) for SARS-CoV, where the short conformation is found in thinner regions of membrane as opposed to the long form.

### Membrane compression, M protein aggregation, and spontaneous membrane curvature

**Fig. 7.**
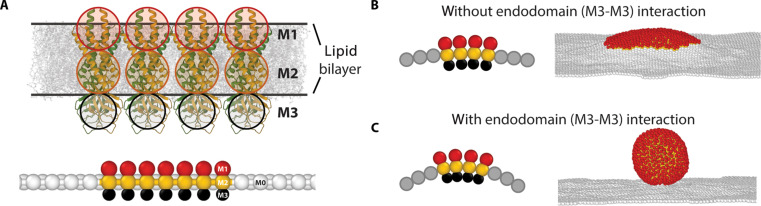
Coarse-grained simulations of M proteins embedded in a flat membrane. (**A**) Schematic illustration of M proteins embedded in lipid bilayers. The top figure shows the actual size of the lipid bilayer and M proteins [Protein Data Bank (PDB): 7VGR (*1*)], and the bottom figure shows the coarse-grained model used in the simulation. (**B**) Schematic figure (left) and simulation snapshot (right) for the case that the M proteins have no endodomain interactions (ϵ_M3-M3_ = 0). The M proteins aggregate and form a small bump due to the line tension, but no further budding is observed in the simulation. (**C**) Schematic figure (left) and simulation snapshot (right) for the case that M proteins have endodomain interactions (ϵ_M3-M3_ = 8*k*_B_*T*). The interaction helps M proteins overcome the energy barrier and the membrane buds into a spherical shell. For each M protein, the domain particles have a diameter size of 1.0*a* (M1), 1.0*a* (M2), and 0.8*a* (M3), where *a* is the system length unit corresponding to 3 nm. For both simulations, the transmembrane domain M2 interacts with M2 with strength ϵ_M2-M2_ = 4*k*_B_*T*. The membrane is represented as a triangular lattice where rigid particles (M0) occupy the vertices due to the incompressibility of the lipid membrane. The M0 particles interact with M with the strength ϵ_M-M_ = 4*k*_B_*T*. The rest of particles have excluded volume repulsion defined through a cutoff Lennard-Jones potential.

The membrane thickness reduction around M protein observed in Fig. 6 is likely due to the height mismatch between the hydrophobic transmembrane domain and the thickness of the lipid bilayer. From the literature (*12*) and as confirmed with our all-atom MD (fig. S5D), the transmembrane region of the M protein dimer is around 4 nm. The experimentally measured thickness of the lipid bilayer is 4.5 nm as shown in Fig. 4F. Thus, the protein hydrophobic region is ~0.5 nm smaller than the membrane thickness. This hydrophobic mismatch leads to compression of the bilayer in the immediate proximity of the M protein that we show in Fig. 6 (C to E). The thinning of the membrane in the vicinity of the protein results in an energetic cost due to bend and tilt deformations in the lipid layers, which gives rise to an effective line tension (*13*). Using our measurements of the mechanical moduli of the membrane, informed by estimates from literature (*14*) and the magnitude of the thinning, we can estimate the effective line tension to be at least about 0.35 pN (see the Supplementary Materials for details). This value is consistent with estimates and measurements of line tension for lipid domains with comparable height mismatches (*13*, *15*), which control the growth and size of lipid ordered domains (*15*, *16*), suggesting that the line tension can facilitate the aggregation of M into patches. Our coarse-grained simulation (see the next section) shows that this patch formation leads to the spontaneous curvature of the membrane. In addition, all-atom MD simulations of both M protein conformations show the propensity to individually induce membrane curvature, as seen in fig. S11. However, the attractive interaction between the C-terminal endodomains is essential for the membrane budding (Fig. 7). Our coarse-grained ERGIC simulation reveals that SARS-CoV-2 nucleocapsid (N) proteins and RNA can effectively introduce membrane curvature as well, providing insight into the mechanism underlying virus budding and encapsulation (fig. S12).

### Coarse-grained MD simulation of M protein–induced budding

In general, our understanding of virus assembly is restricted by the limitations in the spatial and temporal resolution of experimental images, which hinder our ability to observe intermediate structures and states (*17*–*20*). To address this challenge, coarse-grained simulations have been extensively used, successfully improving our understanding of virus assembly processes (*21*–*27*). Here, we also perform a series of coarse-grained simulations by modeling M proteins and a flat lipid bilayer to illustrate how the inserted M proteins can give rise to virus budding, as shown in Fig. 7A. Each M protein is built from three hard particles (M1, M2, and M3), where M1 and M2 correspond to the transmembrane domain and M3 denotes the endodomain. The membrane is modeled as a triangular lattice with hard particles (M0) occupying the vertices, on account of the incompressibility of the lipid membrane. We note that the thickness of the membrane is implicitly taken into account through the stretching and bending moduli of the triangular network (*28*); see Materials and Methods for more details. Because most of the experiments and all-atom simulations here are performed on the flat membrane, we also use a flat membrane to investigate the role of membrane protein on curvature. The simulation results are shown in Fig. 7 (B and C), where M proteins interact with each other through Lennard-Jones potentials. Figure 7B shows that transmembrane domain interaction (M2-M2) cannot give rise to M protein–induced membrane curvature, leading to the membrane budding even if the budding particle has lower energy. This is mainly due to the presence of the energy barrier required to bend the membrane. Consistent with the results for a curved ERGIC membrane (*28*), we find that an attractive endodomain interaction is necessary under all the conditions examined to overcome the bending energy penalties. Figure 7C shows that, as we increase the M protein endodomain interaction (M3-M3), the membrane with the embedded M proteins can bud and form a spherical shell. We note that N proteins and RNA can also effectively provide the M protein endodomain interactions and help M proteins bud (*28*); see fig. S12. The budding prerequisites could indicate the mechanism of how coronavirus can encapsidate N proteins and RNA complex into an infective particle (*28*).

## DISCUSSION

This paper reports an expression and purification protocol for SARS-CoV-2 M protein using SUMO tags, which enables us and other members of the scientific community to carry out many new experiments to better understand viral protein function and the life cycle of SARS-CoV-2. We have optimized the method to obtain a very high yield of 50 to 100 mg of protein per liter of culture. Furthermore, we developed a method to reliably insert M protein into phospholipid bilayers in vitro, and our cryo-EM images confirm that expressed M protein dimers are inserted homogeneously oriented into LUVs. We can precisely control the M protein concentration by adjusting the protein-to-lipid mass ratio used to create the vesicles. The production of LUVs with adjustable concentrations of inserted M protein constitutes a new experimental platform for studying the behavior and binding properties of vesicles with realistic densities of M protein.

To create a comprehensive picture of the structure and orientation of M protein in the membrane and the proteins’ effect on the surrounding membrane, we deposited the M protein–containing vesicle as a supported membrane on a mica surface that allows high-resolution AFM imaging. This reveals the SBL retains a mixture of homogeneously oriented M protein dimers and multimers. This protocol results in the M protein C terminus being exposed on the surface of the suspended bilayer, allowing measurements of M protein binding to other SARS-CoV-2 structural proteins and viral RNA.

In addition to AFM imaging, we performed atomistic MD simulations of the M protein in the suspended membrane. Our AFM and MD results indicate that the M protein is oriented perpendicular to the plane of the membrane, with the top of the C terminus extending 2.4 nm above the surface of the membrane, while the N terminus is virtually flush with the membrane surface on the lower leaflet. The membrane-inserted dimer has an elliptical cross section with major and minor diameters of 4.8 and 2.3 nm, respectively. In addition, a second population of embedded protein is found with the same height but with diameters of 9.4 and 5.7 nm, corresponding to aggregates of dimers. This orientation of the M protein with the C-terminal endodomain extended above the membrane facilitates its proposed role as an anchoring site for other structural proteins.

A particularly interesting result from our study is the structure of the membrane in the immediate vicinity of the M protein dimer. Both the AFM images and the atomistic MD show a region of decreased membrane thickness surrounding the protein, on the order of 0.5 nm, which gradually returns to the normal thickness over a distance of 12 nm. This thinning is likely due to the mismatch in the width of the hydrophobic band in the transmembrane region of the M protein dimer (~4 nm) and the hydrophobic lipid region of the membrane (~4.5 nm), which induces a line tension in the membrane estimated to be ~0.35 pN. This line tension can drive aggregation of M protein dimers, even in the absence of specific inter-dimer attraction. Our membrane is suspended on a rigid surface, so we cannot directly observe via AFM whether the M protein dimers cause membrane bending, but our atomistic MD simulations show that the dimer induces a persistent fold in the membrane (which may manifest as a concave indentation for a simulation of a larger membrane patch).

To connect the molecular scale M protein properties to the mesoscale formation of a coronavirus from all its constituent parts, we constructed a coarse-grained model of a membrane containing 14% M protein dimers. We then carried out MD simulations on this model to understand the combined effects of the endodomains, line tension, and spontaneous curvature generation in viral budding. Our simulations revealed that budding and spherical virion formation occur only if M proteins have an intrinsic curvature and interact with each other via the endodomains. The experimental and simulation techniques developed in this study to date open the door to rapid progress toward fully elucidating the function of M protein. A full understanding of the role of M proteins in driving the budding and release of the newly formed SARS-CoV-2 virion from the cell can pave the path for achieving alternate ways of disrupting their formation.

## MATERIALS AND METHODS

### Strains and media

For protein expression and purification experiments, we used the commonly used protein expression strains BL21(DE3) (Thermo Fisher Scientific) (*29*, *30*), BL21-AI (Thermo Fisher Scientific), C41(DE3) (*31*), C43(DE3) (*31*), C41(DE3) pLysS (*31*, *32*), and C43(DE3) pLysS (*31*, *32*). Cells were grown in LB Miller [10 g of tryptone (Bacto, Thermo Fisher Scientific), 5 g of yeast extract (Bacto, Thermo Fisher Scientific), 10 g of NaCl (MilliporeSigma), and ultrapure H_2_O (Milli-Q IQ 7000, MilliporeSigma) to 1 liter of medium (*33*, *34*)] or modified TB [20 g of tryptone, 24 g of yeast extract, 4 ml of glycerol (Thermo Fisher Scientific), 0.017 M KH_2_PO_4_ (MilliporeSigma), 0.072 M K_2_HPO_4_ (MilliporeSigma), and ultrapure H_2_O to 1 liter of medium (*35*)].

### Plasmid construction

Plasmids encoding and expressing M with an N-terminal 6xHis tag and with and without SUMO tag fusions were designed in VectorNTI software (Thermo Fisher Scientific) and synthesized de novo by GENEWIZ/Azenta Gene Synthesis service. Constructs were synthesized to be expressed from a T7*lac* promoter (*36*) with a consensus Shine Dalgarno ribosomal binding site and were cloned into plasmid pUC57-kan-GW. A fluorescently tagged variant, M-mCerulean3 (*37*), was designed and produced similarly with C-terminal fluorescent fusion.

### Cell growth and protein expression and purification

Cells were grown identically for all experiments. First, a seed culture was started by inoculation of 25 ml of medium containing kanamycin (25 μg/ml) in a baffled 125 ml of Erlenmeyer flask from freezer stock and allowed to grow overnight at 37°C in a C76 shaking water bath (New Brunswick Scientific). This culture was used to inoculate 1 liter of medium containing kanamycin (25 μg/ml) in a 2-liter baffled Erlenmeyer flask and grown at 37°C in a C76 shaking water bath until the optical density at 600 nm (OD_600_; SmartSpec Plus spectrophotometer, Bio-Rad) reached ~0.5 to 0.6. At this point, the flask was removed from the water bath and transferred to a shaker (Dura-Shaker, VWR) at room temperature. After cooling to room temperature, the appropriate concentration of inducer to effect full induction was added (0.4% w/v l-arabinose for BL21-AI or 1 mM IPTG for all others), and the culture allowed to grow overnight to saturation.

### Quantification of protein expression and microscopy

To quantify expression of M protein in various *E. coli* strains and media, cells transformed with the indicated plasmids expressing variants of M-mCerulean3 were grown as described above. Two milliliters of culture was then collected and centrifuged (10,000*g*, Eppendorf 5424), and the supernatant medium was aspirated away. Cells were then washed with phosphate-buffered saline (PBS) three times and lastly resuspended in fresh PBS. Cells were diluted with PBS such that OD_600_ < 1.0 and distributed to the wells of a 48-well plate (Corning Costar). The plate was placed in a CLARIOstar microplate reader (BMG Labtech), and the OD_600_ and mCerulean3 fluorescence were measured (excitation at 430/20 nm and emission at 480/20 nm). Expression levels reported in Fig. 1 are calculated as mCerulean3 fluorescence divided by OD_600_. Each measurement was performed with three technical replicates over three experimental/biological replicates.

Fluorescence microscopy was performed on a Nikon Ti-E inverted fluorescence microscope with an Andor DU-897 Ultra camera. For imaging of M-mCerulean3 fluorescence, cells were imaged with a 100× total internal reflection fluorescence objective and illuminated with highly inclined 457-nm laser light from a Milles Griot argon laser fitted on a Nikon LU-4A laser launch and using a Chroma filter cube with Z457/10× excitation and ET485/30m emission filters.

### Protein purification

After growth to saturation as described above, the 1 liter of culture was spun down for 30 min at 4300*g* in an Eppendorf 5910Ri refrigerated centrifuge maintained at 4°C, and the resulting supernatant was discarded. Cells were resuspended in 10 ml of lysis buffer [50 mM NaH_2_PO_4_, 300 mM NaCl, and 10 mM imidazole (pH 8.0)] with lysozyme (1 mg/ml; MilliporeSigma) and benzonase nuclease (50 U/ml; MilliporeSigma) and incubated at 4°C for 30 min on a nutating shaker. The cells were flash-frozen in liquid nitrogen, followed by incubation in a room temperature water bath until thawed. This slurry was then centrifuged at 10,000*g* at 4°C for 30 min. The supernatant clarified cell extract (soluble fraction) was collected and stored at −80°C until purification. The pelleted cell debris was then washed three times in inclusion body wash buffer (PBS with 25% w/v sucrose, 5 mM EDTA, and 1% Triton X-100) followed by centrifugation at 20,000*g* for 30 min at 4°C. After the final wash, inclusion bodies were resuspended in denaturing lysis buffer [8 M Urea (MilliporeSigma), 10 mM Tris (Thermo Fisher Scientific), 100 mM NaH_2_PO_4_ (MilliporeSigma), 50 mM 3-(cyclohexylamino)-1-propanesulfonic acid (CAPS; MilliporeSigma), 0.3% *N*-lauroyl sarcosine (MilliporeSigma), and 1 mM dithiothreitol (DTT; MilliporeSigma) (pH 11.0)] with nutation at room temperature for 30 min. This insoluble fraction was then stored at −80°C until purification.

For purification, samples were processed using a Bio-Rad NGC Quest 10 Plus fast protein liquid chromatography apparatus with a 5-ml EconoFit Nuvia IMAC affinity column (Bio-Rad). To purify protein from the soluble fraction, the column was first equilibrated with Wash Buffer A (50 mM NaH_2_PO_4_, 300 mM NaCl, and 20 mM imidazole). The sample was loaded onto the column and washed with five column volumes of Wash Buffer A. The column was then washed with a linearly increasing mixture of Wash Buffer A mixed with Wash Buffer B (50 mM NaH_2_PO_4_, 300 mM NaCl, and 250 mM imidazole) from 3 to 100% Wash Buffer B composition over 10 column volumes. Last, the column was washed with five column volumes of Wash Buffer B. Throughout, the composition of the eluate was monitored by measuring the absorption at 280 nm, and those samples containing the protein of interest were collected and pooled for further processing. To purify protein from the insoluble fraction, the procedure was identical using Denaturing Wash Buffer A [50 mM NaH_2_PO_4_, 300 mM NaCl, 50 mM CAPS, 0.3% *N*-lauroyl sarcosine, and 20 mM imidazole (pH 11.0)] and Denaturing Wash Buffer B [50 mM NaH_2_PO_4_, 300 mM NaCl, 50 mM CAPS, 0.3% *N*-lauroyl sarcosine, and 300 mM imidazole (pH 11.0)]. All purified SUMO-fused proteins eluted as a single peak.

### Protein refolding and dialysis

To refold denatured proteins purified from the insoluble fraction, samples were dialyzed in regenerated cellulose dialysis membrane [3.5-kDa molecular weight cutoff (MWCO), Spectra/Por] over 2 days at 4°C in 10× volume of refolding buffer [20 mM tris and 10% glycerol (pH 8.0)] with periodic replacement of buffer at least four times over the course of dialysis.

### Cleavage of SUMO tags

To remove the SUMO tag, purified protein (after refolding, if necessary) was mixed with an appropriate volume of 10× SUMO protease cleavage buffer [500 mM tris, 2% Igepal NP-40 (MilliporeSigma), 1.5 M NaCl, and 10 mM DTT (pH 8.0)] along with an excess of Ulp1 6xHis-SUMO protease previously purified from the *E. coli* soluble fraction as described above. Digests were incubated in a 30°C water bath overnight.

### Removal of SUMO and SUMO protease for final purification

To remove the cleaved 6xHis-SUMO tags and SUMO protease, protein digests were mixed with 1 ml of HisPur Ni-NTA resin (Thermo Fisher Scientific) and placed on a rotating mixer maintained at 4°C overnight. After incubation, the samples were applied to an empty Poly-Prep chromatography column (Bio-Rad) and the purified proteins collected as the flow-through. If higher concentrations were required for a given application, then these samples were concentrated using a Pierce PES protein concentrator with appropriate MWCO (Thermo Fisher Scientific).

### Quantification of yields and endotoxin levels

During purification, absorbance at 280 nm was measured by the NGC Quest 10 Plus optical detector and yields calculated using extinction coefficients estimated using the ExPASy ProtParam online tool based on protein sequence (*38*). Estimated concentrations were verified using an RC DC protein assay (Bio-Rad). Briefly, 5 μl of protein samples and standards (Protein Standards I and II, Bio-Rad) were added to 25 μl of reagent A′ and mixed in the wells of a 96-well plate (Corning Costar). To this, 200 μl of reagent B was added to each well, mixed, and allowed to incubate at room temperature. After 15 min, the absorbance at 750 nm was measured using a CLARIOstar microplate reader, and the concentrations of the samples determined by comparison to linear regression of 2× dilutions of the protein standards.

Endotoxin levels of purified samples were determined using a ToxinSensor chromogenic LAL endotoxin assay kit (GenScript) according to the manufacturer’s instructions. Samples (16.67 μl), endotoxin standards, and water as a negative control were added to the wells of a 96-well plate (Corning Costar). To this, 16.67 μl of LAL was added to each well and mixed. The plate was incubated at 37°C for 10 min. Reconstituted chromogenic substrate (16.67 μl) was added, mixed, and incubated at 37°C for 6 min. Then, 83.3 μl of color stabilizer 1 was added and mixed, followed by 83.3 μl of color stabilizer 2, and lastly 83.3 μl of color stabilizer 3. The absorbance of each sample was measured at 545 nm in a CLARIOstar microplate reader, and the endotoxin levels of the samples were determined by comparison to linear regression using 2× dilutions of the endotoxin standards.

### SDS-PAGE and Western blots

To perform SDS-PAGE, 100 ng of each purified protein was mixed with an appropriate volume of 2× Laemmli sample buffer (Bio-Rad) with 5 mM β-mercaptoethanol (MilliporeSigma). Samples were heated at 95°C for 5 min to denature and reduce the proteins and were then separated by electrophoresis through 4 to 15% Mini-PROTEAN TGX Stain-Free gel (Bio-Rad) with tris/glycine/SDS running buffer. Gels were subsequently stained with Oriole fluorescent gel stain (Bio-Rad) and visualized on a UV transilluminator 2000 (Bio-Rad).

For Western blot, the total protein concentration was determined using the bicinchoninic acid protein assay kit (Pierce/Thermo Fisher Scientific, Rockford, IL). Protein samples (25 μg) were mixed with 4× lithium dodecyl sulfate (LDS) sample buffer (Life Technologies, CA, USA) and 10× reducing agent (Invitrogen, Carlsbad, CA) and heated for 5 min at 100°C. Samples were loaded in a 4 to 12% SDS-PAGE gel (NUPAGE, Invitrogen) for electrophoretic separation and subsequently electro-transferred to polyvinyldifluoride membrane (Invitrogen, USA). Membranes were blocked with 5% bovine serum albumin in TBST (tris-buffered saline with Tween 20) for 1 hour at room temperature and, afterward, incubated overnight at 4°C in primary antibodies. Membranes were then washed with TBST (three times for 15 min) and incubated with anti-rabbit horseradish peroxidase (111-036-045; Jackson ImmunoResearch; 1:5000) secondary antibody. Membranes were then imaged with a Bio-Rad ChemiDoc system (Bio-Rad, Hercules, CA) and analyzed using ImageJ software (National Institutes of Health). The following primary antibodies were used: SARS-CoV-2 membrane protein antibody (ProSci, 9165; 1:1000), SARS-CoV-2 envelope protein antibody (ProSci, 9169; 1:1000), SARS-CoV-2 N protein (RayBiotech, QHD43423; 1:1000), and SARS-CoV-2 spike protein antibody (Novus biologicals LLC, NB100-56578; 1:1000).

### Protein reconstitution into vesicles

Monodisperse LUVs were extruded using a vesicle extruder. Lipids with composition mimicking that of the ERGIC were dissolved in chloroform with solid concentration of 5 mg/ml. The molar ratio of different lipids (Avanti Polar Lipids Inc., Alabaster, AL) are 1-palmitoyl-2-oleoyl-glycero-3-phosphocholine:1-palmitoyl-2-oleoyl-sn-glycero-3-phosphoethanolamine:1-palmitoyl-2-oleoyl-sn-glycero-3-phosphoinositol (POPI):1-palmitoyl-2-oleoyl-sn-glycero-3-phospho-l-serine:cholesterol= 0.45:0.2:0.13:0.07:0.151. The chloroform solution was dried in a glass vial with gentle N_2_ gas stream and then vacuumed overnight at −30 in Hg at room temperature. The dried lipid mixture was hydrated with biological relevant buffer (150 mM NaCl and 20 mM Hepes (pH 7.2)] with 30-s vortex, before 10 freeze-thaw cycles in dry ice and 37°C. After the final thawing step, the aqueous solution was passed 11 times through a polycarbonate membrane with 100-nm pores (Nuclepore Track-Etch membrane, Whatman, Chicago, IL). The size and zeta potential of extruded membrane were measured with dynamic and phase analysis light scattering (PALS) techniques using a 90 Plus PALS machine (Brookhaven Instruments, Holtsville, NY).

To reconstitute the M proteins into LUV, the concentrated stock solution of *n*-dodecyl-β-d-maltoside (Avanti Polar Lipids Inc., Alabaster, AL) were added to the freshly extruded LUV solution (5 mg/ml) to reach a final concentration of 80 mM. M protein stabilized by Triton X-100 (stock solution: 1 wt % Triton X-100 per every M protein at 2 mg/ml) was next added to the LUV solution at a mass ratio of M/lipid = 1/100, 1/67, and 1/50 after 30 min of incubation. The solution was allowed another 30 min of incubation before addition of 40 mg of wet BioBeads (Bio-Rad, Hercules, CA) per milliliter of LUV solution to slowly remove the detergent over 3 hours. The second and third doses of BioBeads at 40 mg/ml were also added at 3-hour intervals. A final dose of BioBeads was added at a ratio of 380 mg/ml and incubated for another 3 hours. The M-reconstituted LUVs were separated from the solution via centrifugation at 13,000 revolutions per min for 30 min in a microcentrifuge tube.

### Preparation of SBL samples

To prepare SBL on mica for AFM imaging, 75 μl of LUV solution collected from the bottom of the microcentrifuge tube was added onto freshly cleaved pristine mica and incubated for 20 min to 1 hour for different coverage. The sample was then gently rinsed with 5 ml of buffer. Caution was taken not to expose the SBL sample directly to air. The samples were kept submerged in aqueous buffer until imaging in an AFM fluid cell. Imaging was performed within 1 hour of the sample preparation.

### AFM imaging, force profile collection, and analysis

AFM height images were obtained inside a fluid cell in tapping mode using a MSNL cantilever (Bruker, Camarillo, CA). Images were taken in 512 × 512 line resolution over areas ranging from 250 nm by 250 nm to 1 μm by 1 μm. The effective tip size was calibrated with 10-nm gold nanoparticles (Ted Pella, Redding, CA) using our previously reported protocol 2. Membrane structure was probed with nanoindentation to obtain force profiles. The force profiles were collected in force volume mode with a 16 × 16 sampling resolution over the designated area of interest of 500 nm by 500 nm. To probe the continuum elastic property of the membrane and protein patches, an MLCT probe (Bruker, CA) with a larger calibrated tip radius of 23 nm was used. The area of interest was identified from prior tapping mode imaging. The spring constant of the larger tip radius cantilevers was calibrated to be 0.15 N/m using its thermal oscillation spectrum.

AFM image analysis were performed in Mountains SPIP software (Digital Surf, France). Protein particles were identified by setting the height of SBL top surface as the threshold and only identifying out protrusions above the membrane threshold. The software-identified protrusions were then manually examined to exclude noise and false identification of the protein particles. The false identifications by the software can be ruled out as they are 1- to 2-pixel-wide noise peaks that are much smaller than the proteins that are greater than 40 pixels depending on whether they are isolated protein dimers or large patches.

### Cryo-EM data collection and analysis

Cryo-samples were prepared in a Vitribot Mark IV (Thermo Fisher Scientific). For each sample, Quantifoil grid R2/1 was glow-discharged before a 3.5-μl aliquot was applied and blotted for 4 s at 95% humidity at room temperature. The samples were then plunged into liquid ethane and preserved in liquid nitrogen. The cryo-EM micrographs were obtained using a Talos Arctica (FEI) operating at 200 kV equipped with a Falcon 4i Direct Electron Detector (Thermo Fisher Scientific). The imaging was performed at ×150,000 magnification with a resolution of 0.95 Å/pixel and a defocus range of −1.5 to −2.5 μm. About 150 to 250 images were collected for each sample using automated data acquisition using EPU software (Thermo Fisher Scientific). Images were motion-corrected within EPU. The dataset was processed using Relion software. Approximately 500 areas of interest were manually picked to generate the 2D class average images.

### All-atom MD simulation

All-atom MD simulations were performed using the CHARMM36m force field with the MD package GROMACS, version 2022.3 (*39*, *40*). The CHARMM-GUI input generator was used to set up the simulated systems with periodic boundary conditions and supplied the six steps used for equilibration (*41*–*49*). After equilibration, each system was simulated for 1 ms with a time step of 2 fs in the NPT ensemble. System temperature was maintained at 303.15 K using the Nose-Hoover thermostat (*50*, *51*), with the pressure maintained semi-isotropically at 1 bar in the *xy* dimensions and separately in the *z* dimension using the Parrinello-Rahman barostat (*52*, *53*). The coordinates for each simulation were saved once every 50,000 time steps or every 0.1 ns for a total of 10,000 frames.

Three different membrane systems were simulated: one for each form of the M protein dimer (short versus long) and one as a control only consisting of a membrane. For the simulations involving an M protein dimer, the protein was inserted into the membrane with an orientation and depth that matched other studies (*1*, *2*). Figure S2 shows the schematics for these membrane simulations. In the case of the short form, only residues 9 to 204 were considered [Protein Data Bank (PDB): 7vgs], while residues 9 to 206 were included in the long form structure (PDB: 7vgr) (*1*). The membrane was composed of 15% cholesterol, 45% 1,2-dioleoyl-sn-glycero-3-phosphocholine (DOPC), 20% 1,2-Dioleoyl-sn-glycero-3-phosphoethanolamine (DOPE), 7% 1,2-dioleoyl-sn-glycero-3-phospho-L-serine (DOPS), and 13% 1-palmitoyl-2-oleoyl-sn-glycero-3-phosphoinositol (POPI) in both leaflets, with the solvent consisting of NaCl at a concentration of 0.15 M and transferable intermolecular potential with 3 points (TIP3P) water. Each simulation, depending on the inclusion of a protein, consisted of a 25 nm–by–25 nm membrane in the *xy* plane with at least 5 nm of solvent above and below the protruding protein, yielding a total unit cell thickness of ~18 nm in the *z* dimension.

For runs involving an M protein, the final trajectory was reoriented frame by frame such that the protein was centered in the box for all frames. While each trajectory was fitted to eliminate protein translation across the membrane, this was not the case in the direction normal to the membrane and for the rotation of the protein. To analyze the processed trajectory, the python library MDAnalysis was used (*54*, *55*). Images of these systems were generated using ChimeraX (*56*).

In addition, all-atom simulations for each conformation of the M protein dimer in solvent were performed to compare protein stability with the membrane simulations. These systems involved a box size of approximately 15(16) nm by 15(16) nm by 15(16) nm for the short(long) conformation. The same process and conditions as the membrane simulations were used, with slight deviations in the equilibration steps due to the lack of a membrane.

### Coarse-grained simulation

The coarse-grained simulation is performed using HOOMD-blue package (*57*) and BondFlip plugin (*28*). For the membrane, we initially assign a 100*a*–by–84*a* triangular lattice plane and place M proteins in the middle disk area with a radius of 15*a*. The dynamics of the membrane is modeled through Langevin integrator with *dt* = 0.001 s at room temperature. In every 100 steps, we apply Monte-Carlo bond flip to change the local connectivity of the membrane, where each bond has stretching and bending energy with spring constant 20*k*_B_*T*/*a*^2^ and bending rigidity 20*k*_B_*T* (*28*). We note that each simulation is run for 10,000 s, and a height constraint [*h*(*t*) = 0] is applied for the region with radial distance *r* > 35*a* through the simulation time. The simulation is performed on NVIDIA GeForce RTX 3090, and the visualization is performed with OVITO software (*58*).

## Supplementary Materials

### This PDF file includes:

Supplementary Methods Figs. S1 to S12 References
